# Optimum Leaf Removal Increases Nitrogen Accumulation in Kernels of Maize Grown at High Density

**DOI:** 10.1038/srep39601

**Published:** 2017-01-13

**Authors:** Tiening Liu, Rundong Huang, Tie Cai, Qingfang Han, Shuting Dong

**Affiliations:** 1 Key Laboratory of Crop Physi-ecology and Tillage Science in Northwestern Loess Plateau, Ministry of Agriculture/College of Agronomy, Northwest A&F University, Yangling, Shaanxi 712100, China; 2 The Chinese Institute of Water-saving Agriculture, Northwest A&F University, Yangling, Shaanxi 712100, China; 3 State Key Laboratory of Crop Biology, Agronomy College, Shandong Agricultural University, Tai’an, Shandong Province, 271018, China

## Abstract

Increasing plant density is one of the main approaches of achieving higher yields for modern maize crop. However, there exists leaf redundancy for high-density maize, and leaves of the upper canopy shade more competent leaves at the middle strata. In a two-year field experiments, Jinhai5, a semi-compact corn cultivar, was grown at a density of 105,000 plants ha^−1^ grown until 3 days after silking (3DAS), when plants were subjected to removal of the uppermost two leaves (S_2_), four leaves (S_4_) or six leaves (S_6_), with no leaf removal as control (S_0_). We evaluated the effects of leaf removal on N remobilization, photosynthetic capacity of the remaining leaves for N uptake, and N accumulation in kernels. Our present results concluded that, under high plant density, excising the uppermost two leaves promoted N remobilization from vegetative organs to kernels and enhanced photosynthetic capacity for N uptake, leading to an increased N accumulation in kernels (19.6% higher than control). However, four or six uppermost leaves removal reduced N remobilization from stem and photosynthesis for poor N uptake, resulting in 37.5 and 50.2% significantly reduced N accumulation in kernels, respectively.

Increasing plant density is one of the main approaches of achieving higher yields for modern maize crops^1^,^2^,^3^,^4^, which enables plants to use solar radiation more efficiently^5^,^6^. However, high plant density decreases per-plant crop growth rates, enhances intra-plant competition for assimilates, and accelerates young kernels abortion due to limited carbon and nitrogen supply to the ear^7^,^8^,^9^,^10^,^11^.

Kernels are the most active sink for nitrogen (N) assimilates in cereals after flowering. There are two sources of N for kernel development: N uptake from soil by root during grain filling and remobilized N accumulated from vegetative tissue before anthesis^12^,^13^,^14^. N uptake is dependent upon availability of photosynthates produced by leaves^15^,^16^, and it declines due to exacerbated leaf senescence during grain filling^17^, especially under high plant density. Therefore, reduction in N uptake will enhance N remobilization from leaves and stems^18^, and it may constitute one of the major fraction of seed N. However, excessive N remobilization from leaves brings a decline in photosynthetic activity and results in accelerated leaf senescence, which is not favorable for N uptake^19^. Additionally, previous studies have found that there exists leaf redundancy when maize crops were planted at high density^20^,^21^,^22^. Partially removing these organs (leaves or root) may achieve a more reasonable energy allocation^23^.

Leaves of the upper canopy shade more competent leaves at the middle strata^24^, especially under high plant density. Thus, we hypothesized that at high plant density, optimum leaf removal above the ear leaf would (1) increase N remobilization from vegetative organs, (2) enhance canopy apparent photosynthesis for N uptake during grain filling, and (3) obtain higher N accumulation in kernels at physiological maturity. We tested this hypothesis by removing two, four or six uppermost leaves from top of a plant at three days after silking, with no-leaf removal as control, to understand whether higher N accumulation in kernels of maize at high density could be achieved through optimum leaf removal, to provide a theoretical basis for super-high-yield corn cultivation.

## Results

### Effects of leaf removal on N accumulation and distribution in remaining tissues at four days after silking (4DAS)

At four days after silking (4DAS), we conducted the initial N measurements in remaining leaves and other plant organs to make the corresponding calculations (Table 1). Leaf removal resulted in different levels of reduction in total N accumulation compared with control. The total N accumulation was largest for S_0_, and lowest for S_6_. Similarly, we also calculated the N distribution in remaining plant organs. In comparison to control, N distribution to the remaining leaves decreased in leaf removal treatments, while it increased in stem. At 4DAS, we found N distribution was mainly in stem and other leaves, which accounted for more than 70% of total N accumulation (Table 1).

### Effects of leaf removal on N accumulation and distribution at physiological maturity (R6)

In both growing seasons, the highest total N accumulation was found for S_2_ treatment, while that was decreased sharply in S_4_ and S_6_ treatments (Table 2). Relative to the control, plants in S_2_ obtained 14.7% and 11.2% higher N accumulation in 2012 and 2013, respectively. In addition, at R6, when the grain was a main sink, we found the maximum ratio of N distribution was recorded in the grain. The interaction of leaf removal × year had a significant (*P *< 0.001) effect on N distribution in grain at physiological maturity. On average, N distribution in grain of S_2_ was 6.8% higher than that of S_0_, while for S_4_ and S_6_, it was 18.7 and 28.1% lower, respectively. In addition, we also found that leaf removal effects on N distribution into the stem was significant (*P *< 0.001). Excising the uppermost two leaves significantly reduced the N distribution into the stem, while it was increased in four or six leaves removal treatments.

### Effects of leaf removal on N remobilization in vegetative organs and its contribution to grain N

In our experiments, excising the uppermost two leaves increased N remobilization from stem, leading to a higher accumulation of N in grain, while it was decreased markedly in excessive leaf removal treatments (S_4_ and S_6_). On average, the grain N of S_2_ was 19.6% higher than that of S_0_, while for S_4_ and S_6_, it was 37.5 and 50.2% lower, respectively. In addition, the proportion of remobilized N in grain was also significantly (*P *< 0.001) affected by different levels of leaf removal. We found that more than 50% of N that accumulates in maize grain is derived from mobilization from vegetative organs, and the ratio of remobilized N to grain was significantly higher in leaf removal treatments than control (Table 3). For S_6_, it even reaches to 84.3 and 82.9% in 2012 and 2013 growing seasons, respectively.

### Effects of leaf removal on canopy apparent photosynthesis (CAP)

**Figure 1:**
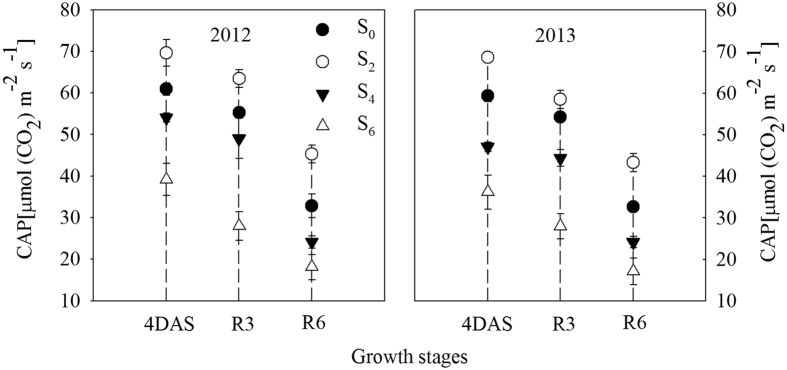
Effects of leaf removal on canopy apparent photosynthesis (CAP) of Jinhai5 grown at high density during 2012 and 2013 growing seasons. S_0_ refers to control (no leaf removal); S_2_, S_4_ and S_6_ refer to the removal of two, four or six leaves, respectively, from top of a plant. 4DAS, R3 and R6 represent four days after silking, milking stage and physiological maturity, respectively. Data are means ± SE (n = 3).

During the two growing seasons, excising the uppermost two leaves made the plants maintain longer duration of high CAP since leaf removal treatments imposed. The highest CAP was found for the S_2_ treatment, while that decreased in the S_4_ and S_6_ treatments (Fig. 1). On average, excising the uppermost two leaves increased CAP at physiological maturity (R6) by 13.5% compared to the control, while it decreased by 26.2% in S_4_ and 46.2% in S_6_, respectively.

### Effects of leaf removal on leaf area index (LAI)

**Figure 2:**
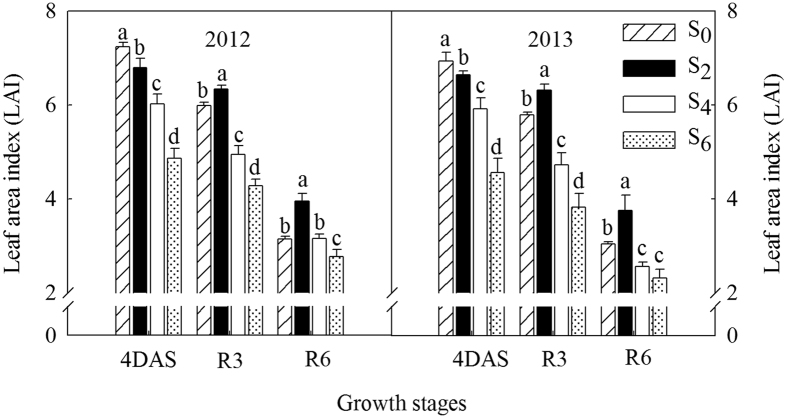
Changes in leaf area index (LAI) as affected by leaf removal under high plant density in different growing seasons from 2012 to 2013. S_0_ refers to control (no leaf removal); S_2_, S_4_ and S_6_ refer to the removal of two, four or six leaves, respectively, from top of a plant. 4DAS, R3 and R6 represent four days after silking, milking stage and physiological maturity, respectively. Data are means ± SE (n = 3). Different small letters in each group indicate significant differences at *P *< 0.05.

Increased levels of leaf removal (S_4_ and S_6_) were accompanied by large decreases in LAI (Fig. 2). However, plants in S_2_ displayed a longer duration of green leaf area in comparison with the control. For instance, excising two leaves led to an increase in LAI at R6 by 26% and 24% in 2012 and 2013, respectively, indicating that leaf senescence in S_2_ was delayed.

### Correlation analysis

**Figure 3:**
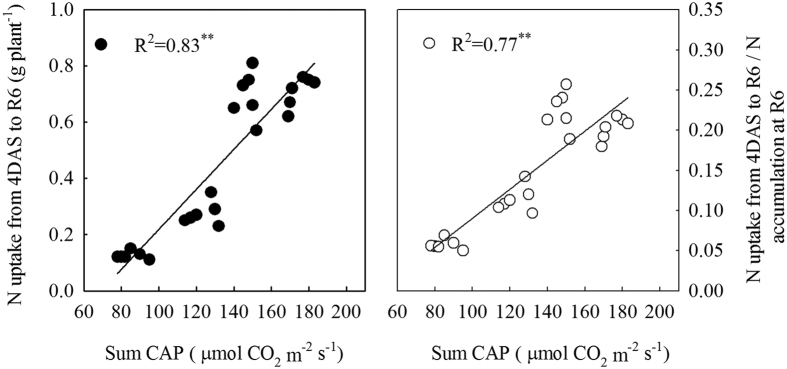
Relationship of sum canopy apparent photosynthesis (CAP) to N uptake from 4DAS to R6 and N uptake from 4DAS to R6 / N accumulation at R6. 4DAS and R6 represent four days after silking and physiological maturity, respectively. Correlation coefficients (r) are calculated and asterisks (**) represent significance at the 0.01 probability (n = 24).

**Figure 4:**
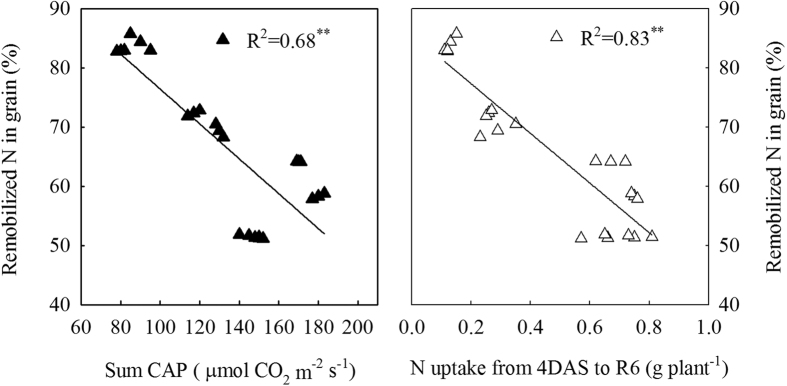
Relationship of remobilized N in grain N at R6 to sum canopy apparent photosynthesis (CAP) and N uptake from 4DAS to R6. 4DAS and R6 represent four days after silking and physiological maturity, respectively. Correlation coefficients (r) are calculated and asterisks (**) represent significance at the 0.01 probability (n = 24).

We found that N uptake from 4DAS to R6 (the difference between N accumulation at R6 and at 4DAS; values from Table 1 and Table 2) and N uptake from 4DAS to R6 / N accumulation at R6 were significantly (*P* < 0.01) and linearly related (R^2^ = 0.83; R^2^ = 0.77; Fig. 3) to sum canopy apparent photosynthesis (CAP) during the same period. However, remobilized N in grain N (%) has a negative (*P* < 0.01) relationship with sum CAP (R^2^ = 0.68; Fig. 4) and N uptake from 4DAS to R6 (R^2^ = 0.83; Fig. 4).

### Effects of leaf removal on grain yield, kernel number per plant (KNP) and kernel weight (KW)

**Figure 5:**
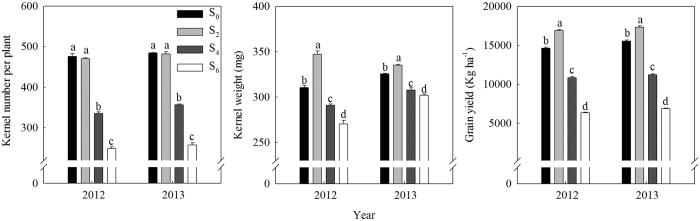
Effects of leaf removal on kernel number per plant (KNP) and kernel weight (KW) at physiological maturity (R6) during 2012 and 2013 growing seasons. S_0_ refers to control (no leaf removal); S_2_, S_4_ and S_6_ refer to the removal of two, four or six leaves, respectively, from top of a plant. Data are means ± SE (n = 3).

In both growing seasons, the highest yield, averaged 17,121 kg ha^−1^, was observed for S_2_ during both experimental years, while lowest yields were recorded in the S_4_ and S_6_ treatments. Relative to the control, plants in S_2_ obtained 15% and 12% higher grain yield in 2012 and 2013, respectively.KNP was greatly reduced by excessive leaves removal (S_4_ and S_6_), whereas no significant difference was found between S_2_ and S_0_. Meanwhile, KW in S_2_ was significantly heavier than that in other treatments (Fig. 5).

## Discussion

N remobilization from leaves and stems constitutes one of the major fraction of seed N^18^. Previous study^25^ reported that N remobilization was totally independent of the ratio of source: sink. Nevertheless, the results of our study revealed that the pattern of total N remobilization and the proportion of remobilized N in grain N were sensitive to different levels of leaf removal imposed at three days after silking (*P* < 0.001; Table 3). Among all the treatments, excising the uppermost two leaves enhanced N remobilization from stem to kernels. Moreover, the proportion of remobilized N in grain N was significant higher in leaf removal treatments than in control, and more than 50% of N that accumulates in maize kernels is derived from mobilization in vegetative organs^26^. On average, it even reaches 71 and 84% for four and six uppermost leaves removal treatments, respectively (Table 3), while the excessive high proportion of remobilized N from the remaining vegetative organs brings a significantly decline in photosynthetic capacity (Fig. 4; R^2^ = 0.68) and is not favorable for N uptake^18^ (Fig. 3; R^2^ = 0.83). According to the present results, we conclude that the reason why the removal of two uppermost leaves was favorable for grain N accumulation might be partially interpreted as the results of an accelerated utilization of N from the vegetative organs for grain growth, especially the stem and leaves (Table 3). Our treatments started at three days after silking, affecting not only the source availability per kernel during the effective grain filling but also when the potential kernel sink capacity is being established^27^. The significantly reduced number of kernels in excessive leaf removal treatments (four or six uppermost leaves removed) re-controlled N remobilization from stem, which partially accounted for the lower N accumulation in kernels (Tables 2 and 3; Fig. 5).

In addition, N uptake during the post-silking period is another major N source for N accumulation in kernels^12^. Several authors have reported that N uptake is dependent upon the availability of assimilates provided by leaf photosynthesis during the post-flowering period^16^,^28^,^29^. Most leaf removal studies often focus on how leaf removal affects the photosynthetic capacity of individual leaves and crop growth rate (CGR) ar^21^,^30^,^31^,^32^. However, maize biomass production is a population-level process under field conditions, and measuring photosynthesis in single leaves will not likely capture the complex process. Moreover, CGR does not reflect the photosynthetic capacity of maize directly. Therefore, we aimed to measure canopy apparent photosynthesis (CAP) performance, which measures the photosynthetic assimilation capacity of maize on a soil area. CAP is the most sensitive and direct reflection on the change of green leaf area caused by different levels of leaf removal in this study^3^,^27^. Within a certain range, the CAP increased with the increase in LAI. However, corn plants under high density with excessively high LAI receive less solar radiation in the middle and lower strata leaves, resulting in accelerated leaf senescence and reduced CAP^4^,^27^. Under high plant density, adequate LAI and an effective blade spatial arrangement are conducive to shaping a canopy structure, which made the plants maintain a higher capacity of CAP^33^. Here, we observed that plants in high density with the two-leaf removal treatment exhibited a longer duration of CAP from when the leaves were removed, whereas higher amount of leaf removal (S_4_ and S_6_) caused rapid reductions in CAP (Fig. 1). This increase in CAP following the removal of two leaves may be related to delayed leaf senescence (i.e. the larger green leaf area; Fig. 2) due to improved light conditions within canopy. Additionally, the lower LAI in S_4_ and S_6_ could partially account for the significantly reduced CAP, indicating that CAP was closely associated with changes in green leaf area. Based on the information of our manuscript, the sum CAP during the post-flowering period was extrapolated from three measurements at the key growth stages, and N uptake during the same period (the difference between N accumulation at R6 and 4DAS; values from Table 1 and Table 2) was also calculated. Our results indicated that post-flowering N uptake variability was significantly and linearly related (R^2^ = 0.83) to the different sum CAP values among defoliation treatments (Fig. 3). Increased photosynthesis increases biomass accumulation, which is partly composed of proteins that contain N. The demand for N is thus increased. When available, that will be taken up by the plant. In addition, N uptake is dependent upon availability of photosynthates produced by leaves, especially the lower layer leaves^15^. Hence, the significantly higher CAPs in two uppermost leaves removal treatment would provide more photosynthates to the roots and increase N uptake during grain filling^16^ (Fig. 3), leading to an increased N accumulation in kernels at physiological maturity (Table 2). In contrast, the significantly reduced CAPs in excessive leaf removal treatments (S_4_ and S_6_) resulted in poor N uptake (Fig. 3), which might be partially related to the root receiving decreased photosynthates, further reducing N accumulation in kernels (Table 2).

Previous research has found that total N accumulation in single plant was decreased when two leaves above ear leaf were cut off ref. 11. However, in our experiments, as observed by Hao *et al*.^34^, proper leaf removal (i.e., excising the uppermost two leaves) made the plants display a higher accumulation of N in grain (19.6% higher than control) and total N per plant (12.9% higher than control; Table 2) under high density. With the intensity of leaf removal, similar to the findings of Rajcan and Tollenaar^35^, N accumulation in kernels decreased by 37.5 and 50.2% in four or six uppermost leaves removal treatments in comparison to control, respectively. Based on the above analysis, the higher kernel N accumulation in S_2_ under high plant density can be explained on the basis of promoted remobilization of N from vegetative organs to grain (Table 3) and enhanced post-silking photosynthetic capacity for N uptake (Figs 1 and 3), and ultimately obtained higher grain yield (Fig. 5), which supported our previous hypothesis. It is noted that the above-mentioned improvement in plant physiological performances upon leaf removal may occur only under high density. The experiment reported in this manuscript was designed on the basis of a preliminary experiment where a series of plant density and leaf removal treatments were combined. In the preliminary experiment, we obtained the highest grain yield in S_2_ at high density (105000 plants ha^−1^), which was significantly higher than S_0_ at optimal density. Interestingly, S_2_ at optimal density resulted in a significant reduction in yield, suggesting that the effect of leaf removal is specific to high density conditions. We therefore focused on evaluating the effect of leaf removal at high density on plant performance from a crop physiological perspective. We recognize that comparing leaf removal effects on plant performance at high density and optimal density will also be quite interesting from a crop physiological perspective, and we intend to conduct these experiments in the future. There are two major contributions to the field of cultivating super-high-yielding corn about this study. Firstly, our results provide a theoretical basis for dense-resistance corn cultivar breeding. We could try our best to breed density-resistance corn cultivar, which has the upmost smaller two leaves. Secondly, we can better control yield by manipulating the development of the uppermost two leaves. An example would be to use chemicals, such as spraying plant growth regulators at the optimum time with the right concentration.

## Conclusion

Excising the uppermost two leaves of corn plants grown under high density conditions (here, 105,000 plants ha^−1^, a relative high plant density in North China Plain) increased N accumulation in kernels at physiological maturity. Such increases were closely related to enhanced remobilization of N from the remaining vegetative organs and promoted canopy apparent photosynthetic capacity for post-silking N uptake. However, severe leaf loss (four or six uppermost leaves removed) imposed close to silking reduced N remobilization from stem and suppressed photosynthesis for poor N uptake, resulting in decreased N accumulation in kernels.

## Materials and Methods

### Experimental design

**Figure 6:**
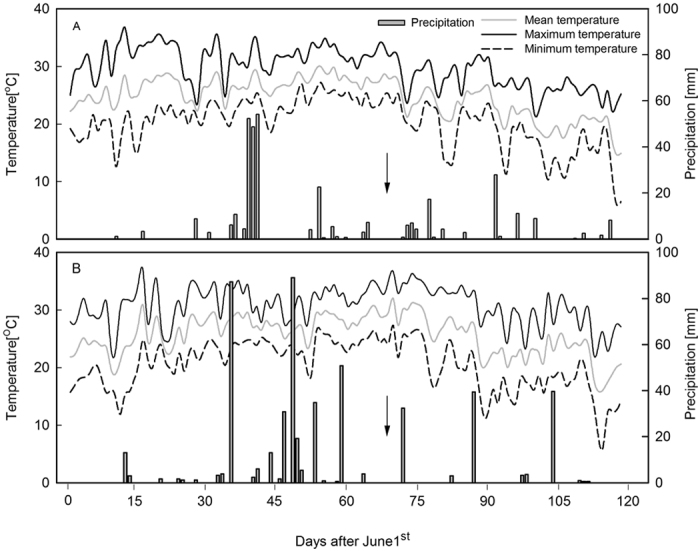
Maximum temperature, minimum temperature, mean temperature (°C) and precipitation (mm) recorded during the growing seasons (from June 1st to September 30th) in 2012 (**A**) and 2013 (**B**). The arrows indicated the timing of leaf removal imposed.

Field experiments were conducted in 2012 and 2013 at the Corn Research Center (36°10′N, 117°09′E, 158 m a.s.l.) of Shandong Agricultural University, Tai’an, Shandong Province, China. The soil type was a sandy loam composed of 11.4 g kg^−1^ organic matter, 0.71 g kg^−1^ total N, 25.6 mg kg^−1^ available phosphate and 107.2 mg kg^−1^ available potassium. All plots were supplied with 120 kg N ha^−1^, 72 kg P_2_O_5_ ha^−1^ and 96 kg K_2_O ha^−1^. All P and K fertilizers and half the N fertilizer were applied at pre-sowing. At the six ligulated leaf-stage^36^ (V6; 13 July 2012 and 15 July 2013), the remaining 60 kg ha^−1^ urea (N 46%) was applied as a top dressing. Precipitation (mm) and the air temperature (°C) were measured by an automatic weather station (climate data, Fig. 6).

**Figure 7:**
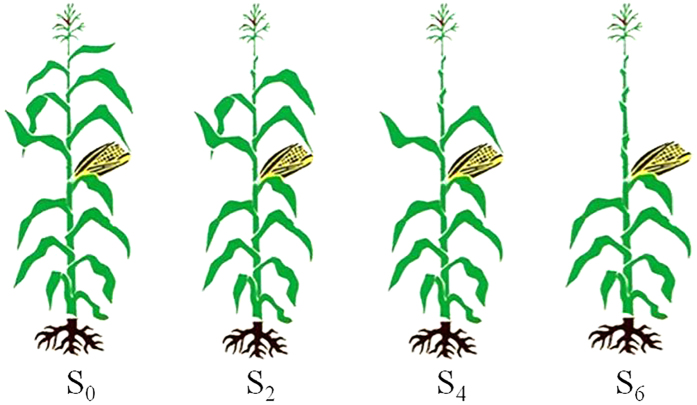
Schematic diagram about how the leaf removal treatments were imposed when Jinhai5 was grown under high density until three days after silking (3 DAS) during 2012 and 2013 growing seasons. S_0_ refers to control (no leaf removal); S_2_, S_4_ and S_6_ refer to the removal of two, four or six leaves, respectively, from top of a plant.

We used the semi-compact summer maize (*Zea mays* L.) cultivar, ‘Jinhai5’ (with six leaves above the ear leaf), in experiments because it is planted widely in local production. Maize seed was overplanted with hand planters on 15 June 2012 and 16 June 2013, and subsequently thinned at three ligulated leaf-stage (V3; 28 June 2012 and 30 June 2013) to a uniform density of 105,000 plants ha^−1^ (a relatively high density for the growing conditions of the North China Plain). Plants were grown until three days after silking (3DAS; 8 August 2012 and 6 August 2013), and then the following treatments by removing different numbers of leaves above the ear leaf were set up (Fig. 7): (1) control, with all leaves left intact (S_0_); (2) the uppermost two leaves removed (S_2_); (3) the uppermost four leaves removed (S_4_); and the uppermost six leaves removed (S_6_). The date of harvest was 7 October 2012 and 5 October 2013.

The experimental design was a random complete block design with three replications. The plot size was 20 m long and 3 m wide with row spacing of 0.6 m, and consisted of 5 rows. The plot was bordered on each side by one guard row of the respective leaf removal treatments, and plants from these lines were not included for any sampling. The irrigation was applied at the stages of tasseling (VT; 5 August 2012 and 3 August 2013) and milking (R3; 27 August 2012 and 26 August 2013) in both growing seasons, and the amount of irrigation was about 75 mm. In the other key growth stages, irrigation was not applied because of adequate rainfall. Disease, weed and pest control followed the agricultural practices of the area.

### Sampling and measurements

#### Sampling

At silking, the healthy, uniform, and representative plants were marked with plastic rope in each plot. Five marked plants in the central three rows of each plot were collected at four days after silking (4DAS; 8 August 2012 and 6 August 2013) and physiological maturity (R6; 7 October 2012 and 5 October 2013), and plants were subsequently dissected into ear leaf, other leaves, stem (including sheath and tassel), cob, bract and grain. All separated components were oven-dried at 80 °C to constant weight. The dry weight of each component was recorded and samples were milled to pass 1-mm screen.

#### N accumulation (g plant^−1^) and distribution in different organs

Nitrogen concentration (g kg^−1^) in each sample was determined using the semi-micro Kjedahl method^37^,^38^. Meanwhile, N accumulation (g plant^−1^) was calculated as the product of dry matter (g) and N concentration (g kg^−1^) in each plant organ. N distribution in different plant organs was calculated as follows:


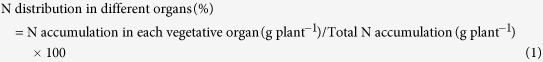


#### N remobilization (g plant^−1^), the proportion of remobilized N in grain N (%) and N uptake (g plant^−1^)

N remobilization (g plant^−1^) from vegetative organs to maize kernels and the proportion of remobilized N in grain N (%) during grain filling were calculated as follows:


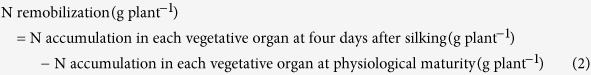



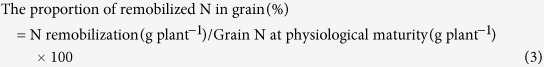



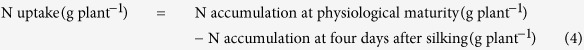


### Canopy apparent photosynthesis (CAP)

Canopy apparent photosynthesis (CAP) was measured in a modified closed gas exchange system using an infrared gas analyzer (GXH-305, China). In order to make the measurements easier and convenient, the aluminum-framed chamber used for measuring CAP consisted of two layers instead of one integral whole chamber, each area of 1.2 × 1 m and 1.4 m in height, which was tall enough to contain plants (the total plant height was 2.39 m) for CAP measurements. The chamber was covered with 0.6-mm-thick Mylar, which permits sunlight to enter at up to 95% of natural intensity. Three battery-powered 60 W fans maintained interior airflow. Before we measured CAP, the one chamber layer was placed to the measured areas, and then the second chamber was placed above the first layer. We then sealed the areas between the below chamber and soil, as well as the areas between the two chambers, with water as soon as possible, and started the measurement. Decreases in CO_2_ concentration were linear and usually measured within 1 min after closing the chamber. Twelve plants in one plot were placed within the chamber, and measurements with three replicates in each treatment were simultaneously taken at four days after silking (4DAS), milking stage (R3) and physiological maturity (R6). CO_2_ exchange rates were expressed on a soil area basis. The canopy photosynthetic rate was calculated as follows^27^:


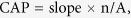


where slope is the decrease in CO_2_ concentration per unit time (μmol mol^−1^ s^−1^), A is the ground area, and n is the number of moles of air in the chamber, calculated as PV / RT, where P is pressure in k Pa, V is volume of the chamber in L, T is the Kelvin temperature K in the chamber, and R is the gas constant, 8.314 k Pa L mol^−1^ K^−1^. In addition, the sum CAP during the post-flowering period was extrapolated from the above three measurements at the key growth stages.

### Leaf area index (LAI)

Three representative plants were selected from the central rows of each plot to determine the green leaf area (GLA) nondestructively at 4DAS, R3 and R6. A leaf was considered to have senesced when half or more of its area had yellowed. Leaf length (L) and maximum width (W) were recorded and used to calculate GLA, i.e. GLA = 0.75 × L × W. Leaf area index (LAI) was calculated as follows: LAI = GLA × N/S, where N is the number of plants within a unit area of land and S is the unit area of land.

### Kernel number per plant (KNP) and kernel weight (KW)

In our experiment, thirty ears were collected from the center three rows of each plot at physiological maturity when black layer formation was complete in all plants. They were used to investigate yield and yield components. Kernel number per plant (KNP) was counted for all the harvested ears. Three samples of 1000 kernels were oven-dried at 80 °C for three days to constant weight, and weighed to estimate kernel weight (KW). All the kernels were air-dried to calculate yield, and grain yield was expressed at 14% moisture.

### Data analysis

Statistical analysis were carried out with SPSS 17.0 (SPSS Institute Inc.). The datas were first checked for normality (Kolmogorov–Smirnov test) and homogeneity of variance (Bartlett–Box test). The datas had a normal distribution and homogeneous variances. Differences among treatments in N remobilization and accumulation were tested by an analysis of variance using least significant difference (LSD) test. At *p *< 0.05, differences were considered statistically significant.

## Additional Information

**How to cite this article**: Liu, T. *et al*. Optimum Leaf Removal Increases Nitrogen Accumulation in Kernels of Maize Grown at High Density. *Sci. Rep.* **7**, 39601; doi: 10.1038/srep39601 (2017).

**Publisher's note:** Springer Nature remains neutral with regard to jurisdictional claims in published maps and institutional affiliations.

## Figures and Tables

**Table 1:** N accumulation (g plant^−1^) and distribution (%) in remaining tissues after leaf removal at three days after silking (3DAS) in 2012 and 2013 growing seasons. S_0_ refers to control (no leaf removal); S_2_, S_4_ and S_6_ refer to the removal of two, four or six leaves from top of a plant, respectively.

Years	Treatment	N accumulation in different plant organs at 4DAS (g plant^−1^)						N distribution in different plant organs at 4DAS (%)				
		Ear leaf	Stem	Other leaves	Cob	Bract	Total	Ear leaf	Stem	Other leaves	Cob	Bract
2012	S_0_	0.15	0.81	1.27	0.20	0.27	2.77	5.11	37.38	40.58	7.99	8.95
	S_2_	0.10	0.72	1.22	0.21	0.22	2.41	4.15	29.88	50.62	8.71	6.64
	S_4_	0.13	0.85	0.83	0.18	0.24	2.13	5.86	42.34	32.88	8.11	10.81
	S_6_	0.12	0.88	0.77	0.19	0.25	2.05	5.85	47.80	27.80	6.34	12.20
	LSD 0.05	0.07	0.05	0.04	0.08	0.07	0.06	0.04	1.48	2.73	0.16	0.57
2013	S_0_	0.15	0.85	1.29	0.22	0.22	2.82	5.70	37.66	40.82	8.54	7.28
	S_2_	0.13	0.73	1.15	0.19	0.19	2.37	5.49	30.80	48.52	8.02	7.17
	S_4_	0.14	0.87	0.81	0.18	0.20	2.14	6.70	39.23	37.32	7.18	9.57
	S_6_	0.15	0.86	0.71	0.19	0.19	2.04	7.35	51.96	29.90	4.90	5.88
	LSD 0.05	0.06	0.03	0.09	0.09	0.06	0.07	0.16	1.69	2.86	0.34	0.37
ANOVA Year (Y) Leaf removal(L) Y × L		ns ns ns	ns * ns	*** *** ns	ns ns ns	ns * ns	* *** ns	* ** ns	ns *** **	ns ** **	* * ns	** * ns
Blocks		ns	ns	ns	ns	ns	ns	ns	ns	ns	ns	ns

Note: 4DAS represents four days after silking. Data represents means ± SE (n = 3). LSD 0.05, least significant difference at 0.05. ns, Not significance; *Significant at the 0.05 probability level; **Significant at the 0.01 probability level; ***Significant at the 0.001 probability level.

**Table 2:** Effects of leaf removal on N accumulation (g plant^−1^) and distribution (%) in remaining tissues at physiological maturity (R6) in 2012 and 2013 growing seasons. S_0_ refers to control (no leaf removal); S_2_, S_4_ and S_6_ refer to the removal of two, four or six leaves from top of a plant at three days after silking, respectively.

Years	Treatment	N accumulation in different plant organs at R6 (g plant^−1^)							N distribution in different plant organs at R6 (%)					
		Ear leaf	Stem	Other leaves	Cob	Bract	Grain	Total	Ear leaf	Stem	Other leaves	Cob	Bract	Grain
2012	S_0_	0.07	0.56	0.75	0.11	0.14	1.56	3.07	2.21	17.66	23.66	3.15	4.10	49.21
	S_2_	0.10	0.51	0.84	0.12	0.15	1.80	3.52	2.84	14.49	23.86	3.41	4.26	51.14
	S_4_	0.06	0.68	0.52	0.08	0.09	0.98	2.42	3.18	28.08	21.38	3.10	3.74	40.52
	S_6_	0.07	0.76	0.40	0.05	0.06	0.83	2.18	3.21	34.86	18.35	2.29	3.21	38.07
	LSD 0.05	0.02	0.04	0.07	0.02	0.02	0.09	0.14	0.25	0.66	0.78	0.15	0.25	2.08
2013	S_0_	0.07	0.56	0.73	0.10	0.13	1.51	3.10	2.39	18.05	23.44	3.16	4.13	48.74
	S_2_	0.10	0.50	0.79	0.11	0.13	1.87	3.49	2.54	14.92	22.17	3.12	3.76	53.49
	S_4_	0.08	0.65	0.55	0.08	0.10	0.94	2.40	3.33	25.08	22.92	3.33	4.17	39.17
	S_6_	0.09	0.81	0.44	0.05	0.07	0.70	2.16	4.17	37.50	20.37	2.31	3.24	32.41
	LSD 0.05	0.03	0.05	0.04	0.02	0.01	0.11	0.19	0.11	0.56	0.31	0.13	0.17	2.69
ANOVA Year (Y) Leaf removal(L) Y × L		ns ns ns	ns *** ns	ns *** *	ns ** ns	ns ** *	ns *** **	* *** ns	* *** **	** *** **	ns *** *	ns * ns	*** ns	ns *** **
Blocks		ns	ns	ns	ns	ns	ns	ns	ns	ns	ns	ns	ns	

Note: R6 means physiological maturity. Data represents means ± SE (n = 3). LSD 0.05, least significant difference at 0.05. ns, Not significance; *Significant at the 0.05 probability level; **Significant at the 0.01 probability level; ***Significant at the 0.001 probability level.

**Table 3:** Effects of leaf removal on N remobilization in remaining tissues (g plant^−1^) and the contribution of remobilized N to grain N in 2012 and 2013 growing seasons. S_0_ refers to control (no leaf removal); S_2_, S_4_ and S_6_ refer to the removal of two, four or six leaves from top of a plant at three days after silking, respectively.

Years	Treatment	N remobilization in different plant organs (g plant^−1^)						Grain N at physiological maturity (g plant^−1^)	Proportion of remobilized N in grain N (%)
		Ear leaf	Stem	Other leaves	Cob	Bract	Total		
2012	S_0_	0.05	0.16	0.47	0.11	0.03	0.80	1.56	51.28
	S_2_	0.06	0.30	0.43	0.13	0.13	1.05	1.80	58.33
	S_4_	0.05	0.17	0.21	0.11	0.15	0.68	0.98	69.39
	S_6_	0.05	0.22	0.17	0.08	0.18	0.70	0.83	84.34
	LSD 0.05	0.01	0.03	0.02	0.01	0.01	0.06	0.09	2.28
2013	S_0_	0.06	0.17	0.42	0.09	0.04	0.78	1.51	51.66
	S_2_	0.09	0.33	0.40	0.16	0.10	1.20	1.87	64.17
	S_4_	0.06	0.22	0.23	0.06	0.11	0.68	0.94	72.34
	S_6_	0.06	0.25	0.17	0.06	0.05	0.58	0.70	82.86
	LSD 0.05	0.02	0.04	0.05	0.02	0.04	0.04	0.11	3.09
ANOVA Year (Y) Leaf removal(L) Y × L		ns ns ns	ns ***^,^*	ns ***^,^ *	ns ** ns	ns ** ns	ns ***^,^**	ns ***^,^**	ns *** ns
Blocks		ns	ns	ns	ns	ns	ns	ns	ns

Note: Data represents means ± SE (n = 3). LSD 0.05, least significant difference at 0.05. ns, Not significance; *Significant at the 0.05probability level; **Significant at the 0.01 probability level; ***Significant at the 0.001 probability level.
